# Books

**Published:** 1882-05

**Authors:** 


### Books.

---

A Study of Tumors of the Bladder. By Alex. W. Stein, M.D., New York. Wm. Wood & Co. 1881.

The author states that four cases of tumor of the bladder had come under his observation in two years; and this lead him to a research among medical journals and other available fields that he might find material for a complete monograph. An extensive bibliographical list gives the sources of his gleanings.

The various tumors are divided first, into benign and malignant; the benign, then, into villous growths, mucous polypi, fibrous polypi, and myomata. The malignant tumors of the bladder may be epithelial, encephaloid, or scirrhus; and, lastly, sarcomatous. All tumors present three symptoms in common—hæmaturia, irritable bladder, and pain. The diagnosis is then considered. The shortness and dilatability of the female urethra, allowing the introduction of the finger, speculum, or other instrument, is favorable to diagnosis. The danger of laceration of the canal is impressed. But both in the male and female, the author admits the great difficulty in some instances of arriving at a conclusion.

The prognosis is not hopeful even in benign growths; it is more favorable, however, in the female than in the male, owing to greater accessibility. A few cases are given in which tumors (mostly polypi), were removed through the female urethra; in several, the vesico-vaginal operation was made. In the male, small tumors have in several instances been removed through the urethra—usually, while crushing a stone. A tumor in the male may be reached by either perineal or supra-pubic cystotomy, the first being most recommended. The higher operation, though, possesses advantages in many cases. In this, the vesical wound must be accurately closed, so as to prevent

*leaking*; but not so in the perineal cut. In malignant growths, operation is but lightly recommended. Free administration of anodynes is enjoined. The author is not enthusiastic as to the efficacy of ergot, gallic acid, and similar remedies in controlling hæmaturia. He prefers cold water irrigation, ice externally or in vagina or rectum, and injecting the bladder with solutions of chloride of zinc, nitrate of silver, iron, alum, etc. As catheterization often irritates and produces hemorrhage, it is desirable to inject through the penis by hydro static or rubber-bag pressure, without the catheter.

---

THE TRANSACTIONS OF THE AMERICAN MEDICAL ASSOCIATION—Vol. xxxii. 1881. Published by the Association. Wm. B. Atkinson, M.D., Phila., Secretary.

This volume, of 684 pages, contains the proceedings and papers of the annual meeting held in Richmond in 1881. The proceedings proper present nothing of unusual interest. The address of the president, Dr. John T. Hodgen, of St. Louis, is a moderately good paper on Some Thoughts in Surgery. He leans toward conservatism, and deplores the tendency of some members of the profession to "run into the ground" any new operation. He urges the importance of a specialist being well versed in general medicine. He urges the early removal of tumors that may prove malignant or semi-malignant. He brings forward a number of other good points, but in a somewhat rambling way.

We have not the space to review each paper presented. Some of these are good, but on the whole not what they should be, as representing the profession of the United States.

In the address on medicine, Dr. Wm. Pepper, chairman of the section, attaches some importance to the administration of large doses of bichloride of mercury in diphtheria. To a child, apparently moribund, 1-32 of a grain was given every second hour with the best results. Dr. W. C. Wile, of Connecticut, urges the value of blood letting in pneumonia. Dr. E. I. Atkinson, of Maryland, has a paper, "May iodide of potassium excite Bright's disease?" He thinks its effects on the kidneys not permanently serious. Dr. Henry O. Marcy, of Massachusetts, presents a double drainage and injection elastic rubber tube or catheter for the bladder.

One of the most notable papers is by Dr. Hunter McGuire, of Virginia, on operative interference in gunshot wounds of the peritoneum. Among his conclusions he says, "I venture to urge, first, the adoption of the proposition of Legouest for operative interference in gunshot penetrating wounds of the peritoneum with intestinal injury; and, secondly, I would add, for operative interference with lesion of any of the contained viscera; and in simple gunshot penetrating wounds without visceral injury. The wound in the abdominal wall should be enlarged, or the linea alba opened freely enough to allow a thorough inspection of the injured parts. Hemorrhage should be arrested. If intestinal wounds exist they should be closed with animal ligatures, trimming their edges first if they are lacerated and ragged."

In the report on necrology we find an interesting sketch of the life of the late W. J. Harrell, M.D., of Bainbridge, Ga. This is from data furnished by Dr. T. S. Hopkins, of Thomasville.

There are a number of very good papers in the volume not mentioned in this hasty review. These, in great part, are the work of specialists. Nor do we say this in a derogatory spirit; for while the methods of some specialists is open to severe criticism, yet it cannot be denied that no little of the advance in medical science is attributable to their labors.

